# Targets for adapting intravenous iron dose in hemodialysis: a proof of concept study

**DOI:** 10.1186/s12882-017-0513-x

**Published:** 2017-03-20

**Authors:** N. O. Peters, N. Jay, J. Cridlig, G. Rostoker, L. Frimat

**Affiliations:** 1 Department of Nephrology, University hospital, Vandoeuvre les Nancy, France; 2 Department of Medical Informatics, University hospital, Vandoeuvre les Nancy, France; 3 Orpailleur, LORIA UMR 7503, Vandoeuvre les Nancy, France; 4 Department of Nephrology and Dialysis, Claude Galien Hospital of the Ramsay Générale de Santé, Quincy-sous-Senart, France

**Keywords:** Intravenous iron, Chronic kidney disease, Dialysis, Anemia

## Abstract

**Background:**

Intravenous iron is widely used to control anemia in dialysis patients and limits costs related to erythropoiesis-stimulating agents (ESA). Current guidelines do not clearly set upper limits for serum ferritin (SF) and transferrin saturation (TSAT). International surveys such as the Dialysis Outcomes and Practice Patterns Study (DOPPS) showed that this lack of upper limits potentially led nephrologists to prescribe iron infusions even for patients with a high SF. Recent publications have suggested a risk of short- and long-term adverse effects related to iron overload. We conducted a proof of concept study to assess the impact of reducing intravenous iron administration.

**Methods:**

In a prospective 8-month study conducted in a hospital dialysis unit, we assessed the impact of a strategy designed to reduce iron infusions. Instead of the usual strategy targeting 30–50% TSAT irrespective of SF, intravenous iron was administered if and only if TSAT was below 20% and SF below 200 μg/L. Routine practices for ESA remained unchanged: hemoglobin target 10–12 g/dL; ESA delivered monthly and dose corrected by 25% as necessary; ESA discontinued temporarily if hemoglobin >13 g/dL; methoxy polyethylene glycol-epoetin beta generally used. Tests were ordered monthly to monitor hemoglobin. Intravenous iron was administered weekly and ESA monthly. Baseline and 6-month TSAT, SF and hemoglobin levels were compared.

**Results:**

Six-month data were available for 45 patients (31 M/14 F; 67.6 ± 14.0 y; 53.9 ± 85.7 months on dialysis). Patients experienced the following comorbidities: ischemic heart disease (*n* = 29, 44%), diabetes mellitus (*n* = 14; 31%), malignant disease (*n* = 11; 24%), transplantation (*n* = 11; 24%) and severe heart failure (*n* = 6; 13%). The mean weekly dose of iron declined from 77.8 ± 87.6 to 24.4 ± 52.9 mg per patient (*p* = 0.0003). SF decreased from 947.7 ± 1056.4 to 570.7 ± 424.4 μg/L (*p* = 0.0001), and TSAT from 41.5 ± 22.4 to 32.6 ± 13.7% (*p* = 0.01). Hemoglobin levels remained stable (11.13 ± 1.05 vs. 11.00 ± 1.16 g/dL, *p* = 0.54) as did ESA dose (126.4 ± 91.9 vs. 108.2 ± 112.7 μg/28 days, *p* = 0.07).

**Conclusions:**

Our study suggests that a regular hemoglobin level can be maintained using regular ESA doses combined with intravenous iron doses adapted to TSAT and SF thresholds lower than those used in routine practice. This strategy reduces the risk of iron overload.

## Background

Although optimal therapeutic management of anemia in hemodialysis patients is still debated, there is a consensus on avoiding blood transfusions. Treatment of anemia was demonstrated to be correlated with an improvement in the quality of life and a reduction in morbi-mortality [1–4]. Some studies, however, highlighted a potential for harm from an overtreatment of anemia by showing that too high target hemoglobin levels were associated with an increased cardiovascular risk [5, 6].

Recommendations for the use of iron and erythropoiesis-stimulating agent (ESA) infusions are not unanimous [7–15]. The choice of biomarkers to monitor iron supplementation, as well as the target levels remains a matter for debate (Table 1). The use of intravenous (IV) iron in dialysis patient has increased substantially since the early 2000s [16–19]. This increase was based on results from clinical studies showing a greater ESAs responsiveness in patients receiving iron supplementation [20–23]. The financial criterion has remained, up until recently, a determinant for IV iron prescribing as shown by escalating trends in its utilization following changes in financial contribution to the treatment of anemia decided by the American Health System in the early 2010s [17]. In addition to its effectiveness for treating anemia, other benefits of IV iron supplementation, particularly cardiovascular, may have contributed to the rise of its prescribing [24–26]. In this context, the lack of an agreement about the level of an upper limit for serum ferritin in international guidelines may be partly responsible for the acceptance of some very high values for serum ferritin by nephrologists.

**Table 1:** Clinical practice guidelines for anemia management in chronic kidney disease according to transferring saturation, serum ferritin and upper limit of serum ferritin for iron supplementation

Guidelines		TSAT(%)	Serum ferritin (μg/L)	Upper limit of serum ferritin (μg/L)
KDOQI 2006 [16]		<20	<100	500
CSN 2008 [18]	HD	<20	<200	None
	PD/ND	<20	<100	None
JSDT 2008 [11]		<20	<100	500
ERBP 2009 [17]		<20	<100	500
KDIGO 2012 [7]		<30	<500	500 (or TSAT > 30%)
CSN 2012 [10]		<30	<500	None
ERBP 2013 [8]	No ESA/no anemia	<20	<100	500
	No ESA/anemia/ND	<25	<200	500
	No ESA/anemia/HD or PD	<25	<300	500
	ESA/anemia/on dialysis	<30	<300	500
KDOQI 2013 [9]		<30	<500	None
NICE 2015 [12]		<20	<100	800

*KDOQI* Kidney Disease Outcomes Quality Initiative, *CSN* Canadian Society of Nephrology, *JSDT* Japanese Society of Dialysis Therapy, *KDIGO* Kidney Disease Improving Global Outcomes, *ERBP* European Renal Best Practice, *NICE* National Institute for Health and Care Excellence, *HD* on hemodialysis, *PD* peritoneal dialysis, *ND* not on dialysis, *ESA* erythropoiesis-stimulating agent, *TSAT* transferrin saturation

Since 2000, several studies have raised concerns about a long-term iron supplementation with supra-physiological doses in renal insufficiency. Thus mortality rate was shown to be correlated with iron dose in the Dialysis Outcomes and Practice Patterns Study (DOPPS) [27]. Several prospective studies demonstrated potential adverse effects due to prolonged use of high dose IV iron [4, 28–32]. Liver iron overload was demonstrated in almost all hemodialysis patients receiving IV iron using magnetic resonance imaging techniques [33, 34]. In addition, several cases of anaphylaxis associated with IV iron infusions were reported [35, 36]. Although it is very difficult to ascertain the frequency of these severe reactions associated with IV iron, it was estimated at one reaction for every five million doses from US data [35]. In addition, three deaths per year were ascribed to intravenous iron infusions in US death certificate data [35].

As a result, the use of IV iron was regulated by the French and European Health Authorities [37, 38]. Taking into account of these recent data, experts have become more cautious about iron supplementation in dialysis [39–41].

In this context, we expressed a desire to change our IV iron supplementation practices by switching from widely prescribing that was presumed to be safe until now to responsible prescribing. Thus we have restricted IV iron supplementation to patients with both serum ferritin levels under 200 μg/L and transferrin saturation (TSAT) under 20%. The change in our IV iron supplementation practices for the management of iron deficiency in hemodialysis patients has been systematically implemented in our dialysis unit since February 2014.

## Methods

### Study design

We conducted a prospective 8-month study to assess the efficacy and the safety of this approach that was more restrictive in iron supplementation for the management of anemia among all patients undergoing dialysis in our hospital dialysis unit.

Enrolled patients were all adults who had been on dialysis for at least 3 months in our unit. During dialysis sessions, they were given anticoagulant as an initial single bolus of enoxaparine or a continuous infusion of heparin sodium. Routine practices for ESA remained unchanged: hemoglobin target levels were 10–12 g/dL; ESA was delivered monthly and its dose could be adjusted by 25% as necessary; ESA was discontinued temporarily if hemoglobin was above 13 g/dL; methoxy polyethylene glycol-epoetin beta was mainly used.

Biochemical parameters for iron status and for the assessment of anemia had been followed in all patients undergoing dialysis in our unit between February and September, 2014.

### Data collection

At the beginning of the follow-up, we collected medical history, treatment and the main biochemical parameters for all patients. We checked comorbidities associated with chronic kidney disease (CKD), such as diabetes, ischemic cardiopathy, cancer, malignant blood disease, and digestive disease, as well as treatment, particularly anticoagulant and antiplatelet agents.

Regarding dialysis sessions, we reported the following parameters: treatment options for renal replacement therapy, such as hemodialysis or hemodiafiltration, weekly duration of dialysis sessions, and ESA dose.

The following biochemical parameters were assessed every 28 days: hemoglobin, TSAT, serum ferritin, serum calcium, serum phosphorus, serum β2-microglobuline, serum albumin and C-reactive protein (CRP). Parathyroid hormone (PTH) level was assessed within the first weeks after the beginning of the study and at the end of the follow-up period. The following patient outcomes had been reported throughout the follow-up: death, transplantation, presence in our dialysis unit after a 6-month follow-up and transfer to another dialysis unit, as well as hospitalizations and reasons of admissions.

### Regimens of IV iron supplementation

At the beginning of the study, the target dose-regimen for IV iron supplementation aimed to maintain TSAT between 30 and 50%, irrespective of serum ferritin levels, in ESA-treated patients in order to avoid iron deficiency anemia. In ESA-naive patients, IV iron supplementation was given in those with hemoglobin level under 12 g/dL or iron deficiency (serum ferritin < 200 μg/L and TSAT < 20%).

Our new therapeutic strategy for iron deficiency therapy aimed to prescribe IV iron supplementation only in anemic patients with TSAT <20% and serum ferritin < 200 μg/L, simultaneously. The weekly iron dose was 100 or 200 mg in patients requiring iron supplementation.

In our unit, iron is administered intravenously as a weekly single dose during the midweek dialysis session in patients on hemodialysis three times per week. Our source of iron is ferric hydroxide sucrose complex.

### Assessment criteria

The main assessment criterion was the change in hemoglobin levels after adaptation of IV iron therapy in patients with a 6-month follow-up in our unit.

The secondary criteria were the change in TSAT, serum ferritin levels and ESA dose after a 6-month follow-up period.

### Statistical analysis

Comorbidities in patients at inclusion, as well as characteristics of treatment and dialysis sessions are expressed in % of patients. Biochemical parameters, iron doses and ESA doses are described by mean and standard deviation. Student’s t-test and McNemar’s test were used to compare change of biochemical parameters and of IV iron prescription during the study, respectively.

## Results

**Fig. 1:**
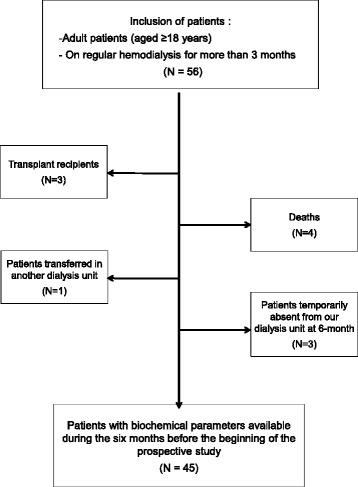
Flow diagram of the study

Among the 55 patients undergoing dialysis in our unit at the beginning of the study, 45 had been followed up throughout the study as shown in Fig. 1.

### Patient characteristics

Sample characteristics are detailed in Table 2. Our population study consisted mainly of men (68.9%). The mean age was 67.6 ± 14.0 years. The mean duration of renal replacement therapy was 53.9 ± 85.7 months. The most common cause of end-stage renal failure was hypertensive nephropathy. Ischemic heart disease and diabetes were the most concomitant pathologies. The majority of patients were given antiplatelet agents.

**Table 2:** Clinical characteristics of sample at inclusion (*N* = 45)

Characteristics	
Gender, N (%)	
Male	31 (68.9%)
Age (years)	67,58 ± 14,02
History of chronic kidney disease, N (%)	
Hypertensive nephropathy	21 (46.7%)
Glomerular nephropathy	7 (15.6%)
Uropathy	5 (11.1%)
Diabetes	4 (8.9%)
Others	8 (17.8%)
Antecedents, N (%)	
Transplantation	11 (24.4%)
Diabetes	14 (31.1%)
Malignant disease	11 (24.4%)
Infection	11 (24.4%)
Hemorrhage	12 (26.7%)
Heart disease	29 (64.4%)
Ischemic heart disease	20 (44.4%)
Heart failure	6 (13.3%)
Previous time of dialysis (months)	53,97 ± 86,26
Type of dialysis, n (%)	
Hemodialysis	32 (71%)
Hemodiafiltration	13 (29%)
Vascular access, N (%)	
Arteriovenous fistula	37 (82.2%)
Arteriovenous graft	3 (6.7%)
Venous catheter	5 (11.1%)
Treatment, N (%)	
Anticoagulants	7 (15.6%)
Antiplatelet agents	32 (71.1%)

Anticoagulation with low molecular weight heparin (LMWH) during dialysis sessions was used in all patients. The mean duration of dialysis session was 13.1 ± 1.7 h per week. Patients were undergoing hemodialysis (68.9%) and hemodiafiltration (31.1%).

### Patients’ outcomes

Patients were followed up during 22.5 patient-years.

Reported reasons for lost to follow-up (transplantation, death, center change or unavailable data) are detailed in Fig. 1. The causes of deaths were attributed to cardiovascular diseases (*N* = 3) and infection (*N* = 1). Temporary absences from our unit were due to transient dialysis in other centers for medical reasons or holidays.

A total of 27 hospital admissions have been recorded during the follow-up, i.e. a hospitalization rate of 1.2 patient-years. Infections were responsible for one out of four admissions.

### Biochemical parameters

**Fig. 2:**
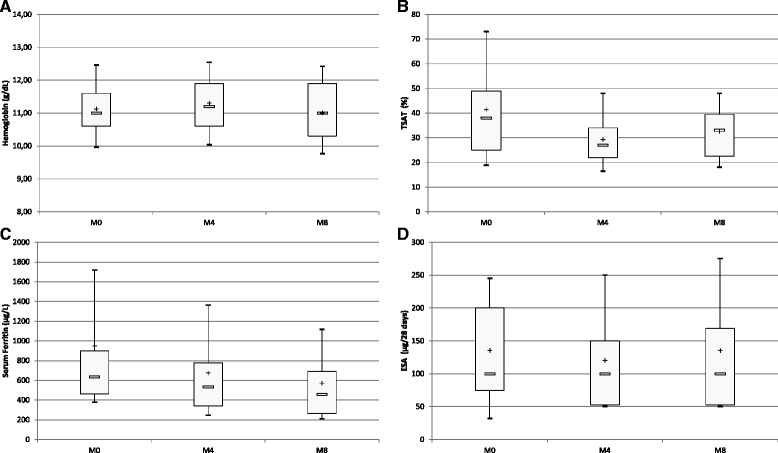
Box-and-whisker plots of change in biochemical indicators of iron status and ESA doses during the follow-up period. **a** Box-and-whisker plots of change in hemoglobin levels during the follow-up period. Limits of boxes are 1^st^ quartile (*lower limit*) and 3^rd^ quartile (*upper limit*), with + and □ being the mean and median values, respectively. Ends of whiskers indicate 1^st^ decile (*lower end*) and 9^th^ decile (*upper end*) (*p* = 0.54). **b** Box-and-whisker plots of change in TSAT during the follow-up. Limits of boxes are 1^st^ quartile (*lower limit*) and 3^rd^ quartile (*upper limit*), with + and □ being the mean and median values, respectively. Ends of whiskers indicate 1^st^ decile (*lower end*) and 9^th^ decile (*upper end*) (*p* = 0.01). **c** Box-and-whisker plots of change in serum ferritin during the follow-up. Limits of boxes are 1^st^ quartile (*lower limit*) and 3^rd^ quartile (*upper limit*), with + and □ being the mean and median values, respectively. Ends of whiskers indicate 1^st^ decile (*lower end*) and 9^th^ decile (upper end) (*p* = 0.0001). **d** Box-and-whisker plots of change in prescribed ESA doses during the follow-up. Limits of boxes are 1^st^ quartile (*lower limit*) and 3^rd^ quartile (*upper limit*), with + and □ being the mean and median values, respectively. Ends of whiskers indicate 1^st^ decile (*lower end*) and 9^th^ decile (*upper end*) (*p* = 0.07)

Hemoglobin levels remained stable throughout the follow-up (*p* = 0.54) (Fig. 2a). An expected significant decrease, by contrast, was observed for biochemical markers of iron status, such as serum ferritin (*p* = 0.0001) and TSAT (*p* = 0.01) (Fig. 2b and c). During the same period, the number of patients with iron deficiency anemia remained stable; the number of patients with TSAT values under 20% decreased from 8 to 7; the number of patients experiencing serum ferritin levels under 200 μg/L quadrupled, without any patient with serum ferritin levels under 100 μg/L. PTH and CRP levels remained stable throughout the follow-up. β2 microglobuline significantly decreased (*p* = 0.00001) and serum albumin levels significantly increased (*p* = 0.0002), as shown in Table 3.

**Table 3:** Comparisons of biochemical parameters and treatment for anemia during the 6-month follow-up

	At inclusion (M0)	At month 3	At month 6	*P*
Biochemical parameters				
Hemoglobin (g/dL)	11.13 ± 1.05	11.30 ± 1.09	11.00 ± 1.16	0.54
TSAT (%)	41.47 ± 22.37	29.27 ± 10.88	32.58 ± 13.63	0.01
Serum ferritin (μg/L)	947.71 ± 1056.42	672.93 ± 490.16	570.73 ± 424.41	0.0001
Parathyroid hormone (pg/mL)	188.03 ± 154.67	ND	212.73 ± 176.85	0.27
β2-microglobuline (mg/L)	25.88 ± 7.77	22.96 ± 5.57	21.80 ± 6.53	0.00001
C-reactive protein (mg/L)	14.59 ± 27.10	9.04 ± 10.14	11.81 ± 30.60	0.64
Serum albumin (g/L)	35.07 ± 4.52	37.34 ± 2.99	40.02 ± 4.58	0.0002
Treatment				
ESA (μg/28 days)	126.4 ± 91.9	109.63 ± 101.03	108.20 ± 112.70	0.07
Iron supplementation (mg/week)	77.78 ± 87.62	15.56 ± 42.40	24.44 ± 52.90	0.0003

*ESA* erythropoiesis-stimulating agent

### Management of anemia

At the beginning of the study, patients were given a mean weekly iron dose of 77.8 ± 87.6 mg - i.e. IV iron infusion of 310 mg per month. Among the 45 patients with a follow-up, 25 patients were given IV iron infusion during dialysis sessions; the mean weekly iron dose of iron was 140.0 ± 70.1 mg – i.e. IV iron infusion of 560 mg per month.

**Fig. 3:**
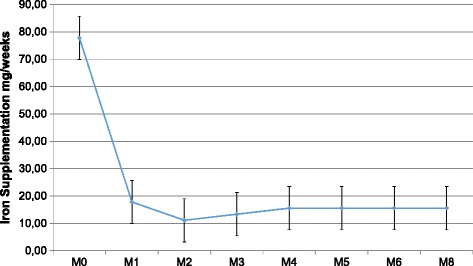
Iron supplementation during the follow-up period

After a 6-month period, the mean weekly iron dose was significantly reduced to 24.4 ± 52.9 mg (*p* = 0.0003) – i.e. IV iron infusion of 97.6 mg per month (Fig. 3). Only nine patients out of the 45 patients were treated with a mean weekly iron dose of 122.2 ± 44.1 mg – i.e. IV iron infusion of 488 mg per month. In addition, the number of patients receiving IV iron infusion was significantly reduced from 50 to 20% (*p* = 0.001).

At the beginning of the follow-up the mean ESA dose was 126.4 ± 91.9 μg/patient/28 days (*N* = 45); 42 patients were treated and were given a mean ESA dose of 135.4 ± 88.4 μg/patient/28 days, while 3 patients did not receive ESA (Fig. 2d). At the end of the follow-up period, the mean ESA dose was 108.2 ± 112.7 μg/patient/28 days (*N* = 45); 36 patients were treated and were given a mean ESA dose of 135.2 ± 110.5 μg/patient/28 days, while nine patients did not receive ESA. ESA dose remained stable during the follow-up (*p* = 0.07). All treatments of anemia are detailed in Table 3.

## Discussion

The present study highlighted that lower targets for serum ferritin and TSAT enabled a reduction of iron supplementation without lowering hemoglobin levels or increasing ESA dose. The targets for serum ferritin and TSAT we used to adapt treatment were in accordance with the current international guidelines for the management of anemia [7, 8]. Biomarkers and targets recommended for anemia management have been regularly revised by Healthcare Institutions, but are still discussed. Although serum ferritine level and TSAT are traditional biomarkers of iron storage in patients with anemia, there are some concerns regarding their relevance [42, 43]. There are alternative indicators of iron metabolism, such as percentage of hypochromic red blood cells, reticulocyte hemoglobin content, soluble transferrin receptor and serum hepcidin. As these serum biomarkers are not routinely measured, we did not assess them in our study. However, it could be interesting to monitor them within the framework of a strategy for lowering IV iron supplementation.

Efficacy of different routes of administration in iron deficiency therapy was compared, and IV iron infusions seemed to be more efficient than oral iron in CKD patients, in both non dialysis [44, 45] and dialysis patients [22, 46]. However, this should be weighed against some potential complications associated with IV iron infusions. The risk of hypersensitivity reactions during IV iron infusions have led the Health Authorities in Europe and in France to recommend new precautions for IV iron supplementation [37, 38]. In particular, IV iron medicines should only be administered in medical institutions with resuscitation facilities in France. In the light of these data, the current guidelines from the Health Societies and Pharmaceutical Manufacturers recommend that oral iron therapy may still be used as the first option in iron deficiency therapy, especially in non-dialysis CKD patients, and IV iron infusion should only be considered in both patients experiencing intolerance and failure to respond to oral iron [7, 8]. Our approach to lower IV iron supplementation should permit to limit the risk of anaphylaxis by reducing the frequency of infusions.

Several IV iron-containing medicines are currently available [40], and each formulation has its own usage instructions. In our unit, an iron saccharose complex is given intravenously at the weekly dose of 100 or 200 mg as a single infusion during the midweek dialysis sessions. Clinical practices for the use of IV iron supplementation in dialysis differ in terms of drugs, dosing frequency, and amount, but at our knowledge, there is no register for all these practices. Results from a retrospective study comparing maintenance infusions of low iron doses to bolus administration of higher and less frequent doses suggested a correlation between the practice of bolus dosing and the risk of infection [30, 47]. More recently, a study was performed to focus on the highest iron amount infused per month without liver iron overload and suggested that the threshold dose should be 250 mg monthly [48].

In our study, reduction in iron amount was more important in the first months of the follow-up (Fig. 2), illustrating a progressive utilization of iron stores in our patients. However, iron amount was significantly different at the beginning versus the end of the study and achieved a stable amount fivefold lower than the previous one used before the implementation of the new approach. The utilization of iron store observed in our study needs to be viewed against the absence of incident dialysis patients who are more likely to experience iron deficiency.

In addition, hemoglobin levels remained in the recommended therapeutic window. We have maintained the biochemical monitoring of our dialysis patients and did not show any compensatory increase of iron amount and ESA dose after a 1-year period.

Our study has some limitations related to the size of our dialysis unit. Our findings need therefore to be confirmed in larger studies including a comparison with a control group. However, our study is the first observational study suggesting that an approach to lower IV iron supplementation is applicable in dialysis settings without increasing ESA dosing and lowering hemoglobin levels.

## Conclusion

Iron plays a central role in the management of anemia of hemodialysis patients and may improve ESA responsiveness. It is widely used nowadays in daily practice in dialysis units, even when ferritin and TSAT levels are high. As IV iron supplementation can induce severe adverse effects, challenges faced by prescribing physicians are especially to achieve the lowest effective dose. Our study suggests that a strategy designed to lower IV iron supplementation can be applied in a wide range of dialysis units without lowering hemoglobin level or increasing ESA dosing in hemodialysis patients. Further prospective larger studies with a control group are needed to validate this approach in hemodialysis patients. Regarding current clinical practices, a moderate IV iron supplementation seems realistic in everyday clinical practice.

## Abbreviations

CKD: Chronic kidney disease
CRP: C-reactive protein
DOPPS: Dialysis Outcomes and Practice Patterns Study
ESA: Erythropoiesis-stimulating agent
IV: Intravenous
LMWH: Low molecular weight heparin
PTH: Parathyroid hormone
SF: Serum ferritin
TSAT: Transferrin saturation
